# Self-rated health and functional capacity in individuals reporting overlapping symptoms of gastroesophageal reflux disease, functional dyspepsia and irritable bowel syndrome - a population based study

**DOI:** 10.1186/s12876-017-0622-9

**Published:** 2017-05-18

**Authors:** Dorte Ejg Jarbøl, Sanne Rasmussen, Kirubakaran Balasubramaniam, Sandra Elnegaard, Peter Fentz Haastrup

**Affiliations:** Research Unit of General Practice, Department of Public Health, University of Southern Denmark, J. B. Winsløws Vej 9A, DK-5000 Odense C., Denmark

**Keywords:** Dyspepsia, Functional capacity, Gastroesophageal reflux disease, Gastrointestinal symptom complexes, Irritable bowel syndrome, Self-rated health

## Abstract

**Background:**

Gastroesophageal reflux disease (GERD), functional dyspepsia (FD) and irritable bowel syndrome (IBS) are common functional gastrointestinal conditions with a significant impact on daily life. The objectives were to analyse general self-rated health and self-reported functional capacity in adults meeting the criteria for GERD, FD and IBS, respectively, and in individuals who meet the criteria for more than one of the conditions.

**Methods:**

A nationwide study of 100,000 individuals aged 20 years and above, randomly selected in the general Danish population. A web-based questionnaire survey formed the basis of this study. Questions regarding FD and IBS were extracted from the ROME III adult questionnaire. Questions regarding GERD were developed based on the Montreal definition. Self-rated health and functional capacity was measured by single global questions.

**Results:**

Respondents meeting the criteria for either GERD, FD or IBS have significantly higher odds of reporting poor self-rated health and impaired functional capacity compared to individuals not experiencing these functional gastrointestinal conditions. Furthermore, respondents with overlapping gastrointestinal (GI) symptom complexes have significantly higher odds of reporting poor self-rated health and impaired functional capacity compared to respondents with symptoms compatible with only one of the symptom complexes.

**Conclusions:**

This study demonstrates that individuals experiencing symptoms of GERD, FD or IBS report poor self-rated health as well as impaired functional capacity. The impact on self-rated health and functional capacity is highest among individuals experiencing overlapping symptoms of GERD, FD and IBS.

**Electronic supplementary material:**

The online version of this article (doi:10.1186/s12876-017-0622-9) contains supplementary material, which is available to authorized users.

## Background

Global consensus has been established, and research has operationalised gastrointestinal (GI) symptoms into symptom complexes of gastroesophageal reflux disease (GERD), functional dyspepsia (FD) and irritable bowel syndrome (IBS) [1, 2].

The prevalence of GI symptoms is high in the general population; in a recent study, we found that 20% of the adult Danish population has experienced symptoms of either GERD, FD or IBS within a 4 week period [3]. Furthermore, a significant overlap was seen between the GI symptom complexes, meaning that a substantial number of patients with either GERD, FD or IBS met the criteria for one or both of the other conditions [3].

Previous studies have shown that individuals suffering from either GERD, FD or IBS have a substantially lower quality of life [4–6]. These findings emphasise the importance of the patient’s own assessment. However, the patient’s subjective side of the matter comprises other dimensions than quality of life that could be relevant when assessing the impact of GI symptoms. It has been demonstrated that self-rated health and quality of life are distinct constructs that should be differentiated [7]; when evaluating quality of life, patients give much greater emphasis to mental health than to physical functioning, whereas the opposite is the case for self-rated health [8].

Knowledge of how GI symptom complexes and the overlap of multiple GI symptoms affect the individuals in other dimensions than quality of life is sparse. It has been demonstrated that overlapping GI symptoms affect the evaluation of bodily pain and general health [6]. However, it has not been examined whether functional capacity is impaired in individuals meeting the criteria for more than one of the GI symptom complexes. It is plausible that experiencing numerous symptoms affects both the individual’s self-perceived health and functional capacity negatively, but on the other hand, one could assume that it is the quality of symptoms rather than the quantity of symptoms that influences self-rated health and functional capacity.

Taking the patient’s own assessments into consideration is important not only as a sign of interest and empathy from the clinician; it can also be important to guide consultations to important questions, and to determine which efforts should be made to handle the symptoms [9].

Therefore, the aim of this study was to analyse general self-rated health and self-reported functional capacity in adults from the general population who met the criteria for GERD, FD and IBS, respectively, and in individuals who met the criteria for more than one of the conditions.

## Methods

### The study population

The study was designed as a nationwide cohort study of 100 000 people randomly selected from the general population. All Danish citizens are registered in the Danish Civil Registration System (CRS) with a unique personal identification number. From the CRS, 100 000 adults aged 20 years or above were randomly selected and invited to participate in a survey concerning a broad range of symptom experiences. The individuals received a postal letter explaining the purpose of the study. In the letter, a unique 12-digit login for a secure webpage was included. This provided access to a comprehensive web-based questionnaire. In order to prevent the exclusion of people with no internet access, the participants were offered the opportunity that the survey could be conducted as a telephone interview. The data collected from the questionnaire form the basis of this study.

### The questionnaire

The questionnaire was designed as a comprehensive questionnaire exploring a broad range of symptom experiences, including gastrointestinal symptoms. Questions regarding GI symptoms were formed using internationally validated scales. The questions regarding FD and IBS were extracted from the ROME III Adult Questionnaire (RIIIAQ), which is validated by the ROME foundation [2]. The RIIIAQ was translated into Danish according to standardised methods [10]. The questions regarding GERD were developed by an expert panel, comprising researchers in the gastroenterological field, on the basis of the Montreal definition and with inspiration from a prior Danish study [1, 11]. The questionnaire was evaluated regarding comprehensibility, relevance, acceptability and feasibility, and pilot tested before use. Further details on the design of the study and data collection are described in details elsewhere [12]. See also Additional file 1 including the questionnaire developed for use in this study.

### Defining GERD, FD and IBS

Based on the Montreal definition stating that GERD is defined as a *‘condition that develops when the reflux of the stomach contents causes troublesome symptoms and/or complications’,* we chose to operationalise the definition of GERD with the criteria listed in Table 1. The first criterion includes individuals who have troublesome symptoms based on frequency and severity of reflux symptoms as suggested by the Montreal definition [1], while the second criterion includes individuals who have less frequent symptoms, but who might still find these symptoms troublesome due to an aggravating factor, specified in this study as impaired sleep or impaired daily activities. The latter criterion was included due to a study showing that it is too simplistic to define GERD on the basis of frequency and severity alone [11]. We defined FD and IBS according to the ROME III criteria. Table 1 shows how the diagnosis of FD and IBS has been operationalised by the ROME foundation using the ROME III Questionnaires (RIIIAQ). The ROME III criteria for FD state that, in order to be diagnosed as having FD, there must be no proof of structural diseases likely to explain the symptoms [2]. In the present study population we do unfortunately not have information about whether they did undergo upper endoscopy. A Danish study reported normal endoscopy in 59% of the patients referred with dyspeptic symptoms, and a study from a Brazilian outpatient clinic found 66% of the patients to have functional dyspepsia [13, 14]. We therefore chose to maintain the term functional dyspepsia since this is the most likely diagnosis in an unselected population experiencing dyspepsia. However, we cannot exclude the possibility of individuals in this study having an organic lesion at endoscopy.

**Table 1:** Operationalising the diagnosis of Gastroesophageal reflux disease (GERD), Functional dyspepsia (FD), and Irritable bowel syndrome (IBS) in terms used in the survey

GERD
A condition that develops when reflux of the stomach contents causes troublesome symptoms and/or complications. Must include at least one of the two criteria: • Mild symptoms occurring more than 1 day a week or moderate to severe symptoms at least once a week. • Impaired sleep at least to some extent, or impaired daily activities at least to a lesser extent and mild symptoms occurring at least once a week, or moderate to severe symptoms at least 2–3 times a month.
FD
Must include one or more of the following: • Uncomfortably full after a regular-sized meal more than 1 day/week in the last 3 months. Onset more than 6 months ago. • Unable to finish a regular-sized meal more than 1 day/week in the last 3 months. Onset more than 6 months ago. • Pain or burning in the middle of the abdomen at least 1 day/week in the last 3 months. Onset more than 6 months ago.
IBS
Must include: Pain or discomfort in the abdomen at least 2 to 3 days/month in the last 3 months with onset at least 6 months ago. For women, the pain must not occur only during menstrual bleeding. And at least 2 of the 3 following criteria: • Pain or discomfort gets better after bowel movements at least sometimes. • Onset of pain or discomfort associated with more frequent stools at least sometimes or onset of pain, or discomfort associated with fewer stools at least sometimes. • Onset of pain or discomfort associated with loose stools at least sometimes, or onset of pain or discomfort associated with hard stools at least sometimes.

### Self-rated health and functional capacity

The questionnaire comprised a single global item concerning general self-rated health extracted from the Short Form 36 questionnaire [15]. The question asked the participants to rate their overall health status with the wording: ‘In general, would you say your health is:’. The answering categories were: ‘Excellent’, ‘Very good’, ‘Good’, ‘Fair’ and ‘Poor’. Furthermore, functional capacity was measured by the single global question: ‘Do you feel well enough to do what you feel like doing?’. The answering categories were: ‘Yes, mostly’, ‘Yes, sometimes’, ‘No, almost never’ and ‘I don’t know’. The questions were used in former national population-based studies [16, 17].

### Data analyses

The respondents were divided into two age groups of 20–49 and ≥ 50 years of age. The scale of self-rated health was dichotomised into ‘good’ and ‘poor’ self-rated health. Good self-rated health comprised the answers ‘Excellent’, ‘Very good’ and ‘Good’, whereas the answers ‘Fair’ and ‘Poor’ were categorised as ‘Poor’. The scale of functional capacity was dichotomised into ‘unimpaired’ and ‘impaired’ functional capacity. Unimpaired functional capacity comprised the answers ‘Yes, mostly’ and ‘Yes, sometimes’. The answer ‘No, almost never’ was defined as impaired functional capacity. For individuals answering ‘I don’t know’, we were unable to classify their functional capacity, and their answer was categorised as missing. Chi-square tests were used to test for differences between gender and age groups, respectively.

Logistic regression models were used to test for interaction between gender and age groups for self-rated health and functional capacity because we hypothesised that self-rated health and functional capacity would be reported differently among men and women under and over 50 years of age. The distribution of proportions in categories of self-rated health and functional capacity was calculated for individuals meeting the criteria for GERD, FD, IBS, respectively, and the overlap between criteria.

Logistic regression analyses were used to calculate odds ratios (ORs) with 95% confidence intervals (CIs) for the associations between poor self-rated health, impaired functional capacity and GERD, FD and IBS and the overlap of these, respectively. A subgroup analysis for individuals meeting at least one of the criteria for the GI symptom complexes was carried out. Due to correlation between self-rated health and functional capacity, the analyses were carried out separately for the two outcomes. The ORs were calculated separately for men and women in the two age groups due to interaction. Data analyses were conducted using StataIC 13©.

## Results

**Fig. 1:**
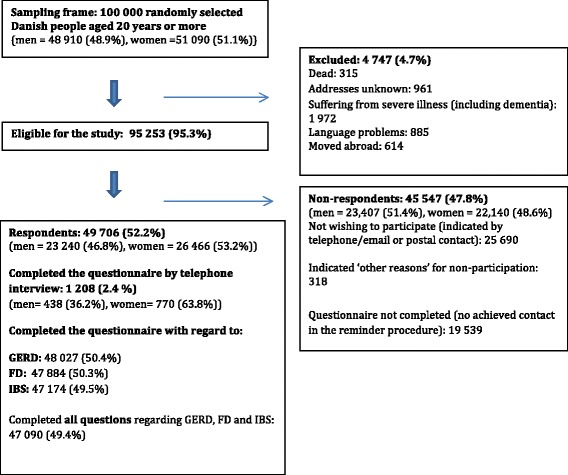
Study cohort

Of the 100, 000 randomly selected subjects, 4474 (4.7%) were not eligible because they had either died, could not be reached due to unknown address, were suffering from severe illnesses (including dementia), had language problems or had moved abroad. Of the 95 253 (95.3%) eligible subjects, 49 706 completed the questionnaire, yielding an overall response rate of 52.2% (Fig. 1). The median age of the respondents was 52 years Interquartile range (IQR) (40–64) compared to 50 years IQR (36–63) for non-respondents. Slightly more respondents were women (53.2%) compared to non-respondents (48.6%). The study group of interest for this paper is a subgroup of 47 090 respondents completing all the questions regarding GERD, FD and IBS, respectively. More women were represented in the age groups 20–49 years and above 50 years, respectively (*p* < 0.001), and significant more respondents were in the age group above 50 years among both gender (*p* < 0.001).

A total of 770 (1.6%) of the respondents met the criteria for all three symptom complexes, Table 2. The distribution of self-rated health and functional capacity of respondents meeting 1) none of the criteria for the GI symptom complexes, 2) the criteria only for GERD, FD and IBS, respectively, and 3) the overlaps GERD + IBS, FD + IBS, GERD + FD and GERD + FD + IBS, respectively, appear from Table 2. All GI-complexes occurred more frequently among females.

**Table 2:** Self-rated health and functional capacity of respondents meeting the different criteria for the GI symptom complexes and overlaps between the criteria

			General self-rated health, n (%)					Functional capacity, n (%)			
	Age, mean (SD)	Women (%)	Excellent	Very good	Good	Fair	Poor	Yes, mostly	Yes, sometimes	No, almost never	I don’t know
All (*N* = 47090)	51.7 (15.5)	24 639 (52.3%)	5771 (12.3%)	16 637 (35.4%)	19 003 (40.5%)	4733 (10.1%)	793 (1.7%)	37 656 (80.2%)	7018 (15.0%)	1947 (4.2%)	316 (0.7%)
Non GERD/IBS/FD *N* = 37 137 (78.9%)	51.9 (15.5)	18 739 (50.5%)	5406 (14.6%)	14 508 (39.2%)	14 241 (38.5%)	2559 (6.9%)	318 (0.9%)	31 399 (84.8%)	4463 (12.1%)	953 (2.6%)	219 (0.6%)
Only GERD *N* = 2823 (6.0%)	52.7 (14.8)	1479 (52.4%)	103 (3.7%)	639 (22.7%)	1452 (51.7%)	525 (18.7%)	91 (3.2%)	1924 (68.5%)	662 (23.6%)	198 (7.1%)	26 (0.9%)
Only IBS *N* = 2741 (5.8%)	46.7 (15.2)	1790 (65.3%)	126 (4.6%%)	729 (26.7%)	1345 (49.3%)	462 (16.9%)	67 (2.5%)	1888 (69.2%)	642 (23.5%)	178 (6.5%)	21 (0.8%)
Only FD *N* = 1338 (2.8%)	53.5 (16.3)	759 (56.7%)	81 (6.1%)	337 (25.3%)	585 (43.9%)	261 (19.6%)	68 (5.1%)	870 (65.3%)	301 (22.6%)	147 (11.0%)	14 (1.1%)
GERD + IBS *N* = 790 (1.7%)	51.2 (14.8)	472 (59.7%)	18 (2.3%)	120 (15.2%)	391 (49.6%)	213 (27.0%)	46 (5.8%)	442 (56.1%)	250 (31.7%)	91 (11.6%)	5 (0.6%)
FD + IBS *N* = 610 (1.3%)	49.1 (15.5)	409 (67.0%)	12 (1.9%)	111 (18.3%)	266 (43.9%)	169 (27.9%)	48 (7.9%)	330 (54.5%)	173 (28.6%)	98 (16.2%)	5 (0.8%)
GERD + FD *N* = 881 (1.9%)	54.8 (13.7)	511 (58.0%)	19 (2.2%)	137 (15.6%)	429 (48.9%)	225 (25.7%)	67 (7.6%)	499 (56.9%)	242 (27.6%)	122 (13.9%)	14 (1.6%)
GERD + FD + IBS *N* = 770 (1.6%)	51.8 (14.4)	480 (62.3%)	6 (0.8%)	56 (7.3%)	294 (38.5%)	319 (41.8%)	88 (11.5%)	304 (39.8%)	285 (37.4%)	160 (21.0%)	14 (1.8%)

*GERD* gastroesophageal reflux disease, *IBS* irritable bowel syndrome, *FD* Functional dyspepsia

The results of the regression analyses are shown separately for men and women under and over 50 years of age due to interaction between self-rated health/functional capacity and sex and age. Respondents with any of the GI symptom complexes had significantly higher odds of reporting poor self-rated health and impaired functional capacity compared to respondents with no GI symptoms, Table 3.

**Table 3:** Associations between poor self-rated health, impaired functional capacity and GI symptom complexes

	Total cohort	Men		Women	
		20–49 years	≥50 years	20–49 years	≥50 years
Odds ratios (95% confidence intervals) of poor self-rated health of the total cohort and stratified on age and gender					
Non-GERD/FD/IBS	1	1	1	1	1
Only GERD	3.33 (3.02–3.67)	4.19 (3.31–5.31)	2.84 (2.37–3.41)	3.46 (2.77–4.31)	3.28 (2.78–3.86)
Only IBS	2.85 (2.58–3.16)	3.95 (3.09–5.07)	3.50 (2.80–4.37)	2.59 (2.15–3.12)	2.67 (2.21–3.22)
Only FD	3.89 (3.42–4.44)	3.09 (2.08–4.58)	3.88 (3.06–4.92)	3.92 (2.98–5.17)	4.01 (3.22–4.99)
Two symptom complexes (FD + GERD, FD + IBS or GERD + IBS)	6.07 (5.52–6.67)	6.32 (4.86–8.22)	5.72 (4.75–6.90)	7.69 (6.37–9.27)	5.08 (4.32–5.98)
Three symptom complexes (FD + GERD + IBS)	13.57 (11.71–15.73)	17.01 (11.88–24.36)	12.87 (9.34–17.72)	15.99 (11.90–21.50)	11.47 (8.99–14.63)
Odds ratios (95% confidence intervals) of impaired functional capacity of the total cohort and stratified on age and gender					
Non-GERD/FD/IBS	1	1	1	1	1
Only GERD	2.88 (2.46–3.38)	3.34 (2.24–4.99)	2.75 (2.07–3.67)	3.49 (2.50–4.88)	2.42 (1.82–3.20)
Only IBS	2.65 (2.24–3.12)	3.48 (2.32–5.22)	3.15 (2.22–4.48)	2.57 (1.92–3.44)	2.27 (1.66–3.10)
Only FD	4.72 (3.93–5.67)	2.73 (1.41–5.26)	5.54 (4.05–7.59)	4.24 (2.84–6.34)	4.79 (3.53–6.50)
Two symptom complexes (FD + GERD, FD + IBS or GERD + IBS)	6.05 (5.28–6.93)	6.62 (4.51–9.72)	5.46 (4.18–7.15)	7.67 (5.91–9.97)	5.01 (3.96–6.33)
Three symptom complexes (FD + GERD + IBS)	10.22 (8.49–12.31)	13.43 (8.41–21.46)	11.50 (7.89–16.77)	13.96 (9.80–19.89)	6.50 (4.65–9.10)

Poor self-related health is categorised as ‘Fair’ and ‘Poor’. Impaired functional capacity is categorised as ‘No’, and ‘Almost never’

*GERD* gastroesophageal reflux disease, *FD* functional dyspepsia, *IBS* irritable bowel syndrome

In a subgroup of participants meeting at least one of the criteria for the GI symptom complexes, respondents with overlapping GI symptom complexes had significantly higher odds of reporting poor self-rated health and impaired functional capacity compared to respondents with symptoms compatible with only one of the symptom complexes, Table 4.

**Table 4:** Associations between poor self-rated health, impaired functional capacity and number of gastrointestinal symptom complexes

	Total cohort	Men		Women	
		20–49 years	≥50 years	20–49 years	≥50 years
Odds ratios (95% confidence intervals) of poor self-rated health of the total cohort and stratified on age and gender					
One symptom complex (either GERD, FD or IBS)	1	1	1	1	1
Two symptom complexes (FD + GERD, FD + IBS or GERD + IBS)	1.87 (1.69–2.08)	1.62 (1.22–2.15)	1.76 (1.43–2.17)	2.52 (2.06–3.09)	1.59 (1.33–1.90)
Three symptom complexes (FD + GERD + IBS)	4.19 (3.59–4.88)	4.36 (2.00–6.33)	3.96 (2.84–5.53)	5.25 (3.87–7.13)	3.59 (2.78–4.63)
Odds ratios (95% confidence intervals) of impaired functional capacity of the total cohort and stratified on age and gender					
One symptom complex (either GERD, FD or IBS)	1	1	1	1	1
Two symptom complexes (FD + GERD, FD + IBS or GERD + IBS	1.93 (1.66–2.24)	2.01 (1.32–3.06)	1.57 (1.17–2.12)	2.47 (1.87–3.27)	1.77 (1.36–2.29)
Three symptom complexes (FD + GERD + IBS)	3.27 (2.68–3.97)	4.08 (2.48–6.72)	3.31 (2.23–4.93)	4.49 (3.11–6.49)	2.30 (1.61–3.27)

Poor self-related health is categorised as ‘Fair’ and ‘Poor’. Impaired functional capacity is categorised as ‘No’, and ‘Almost never’

*GERD* gastroesophageal reflux disease, *FD* functional dyspepsia, *IBS* irritable bowel syndrome

The experience of GI symptoms and overlap of symptoms was significantly associated with poor self-rated health and impaired functional capacity for men and women in both age groups.

## Discussion

### Summary of main findings

This study demonstrates that individuals experiencing symptoms of either GERD, FD or IBS have significantly higher odds of reporting poor self-rated health and impaired functional capacity compared to individuals not experiencing these GI symptom complexes. This negative effect is amplified in individuals with symptoms of two or all three of the conditions. The direction of the association is similar for both men and women, irrespective of age.

### Strengths and limitations

A random sample of the general population was provided by means of the CRS register. A response rate of 52.2% was received which is comparable to or higher than other studies investigating self-reported symptoms in a western general population [18, 19]. Slightly more of the respondents were women, and slightly older than the non-respondents [20].

In several studies, self-rated health is estimated by the single question extracted from the Short Form 36, which is a well validated and widely used instrument that has been proved able to predict illness and death [21]. We further added a question about functional capacity to depict the consequences of affected self-rated health for the individual’s everyday living.

Presence of symptoms and affected self-rated health/functional capacity might affect the willingness to respond to this questionnaire. The survey comprised, however, a wide range of symptoms, which might reduce the risk that individuals with GI symptoms would be particularly interested in answering. Overestimation of prevalence estimates cannot be eliminated if individuals with no symptoms at all or with no affection of their self-rated health or functional capacity were less likely to respond. The fact that the questionnaire was web-based might have prevented some individuals from filling in the questionnaire, especially the elderly. However, a possible selection was sought minimised by offering individuals without internet access to complete the survey by telephone interview.

Experience of symptoms within a period of 4 weeks was chosen in order to have a sufficient number of symptom experiences to obtain statistically precise estimates while assuming that people would be able to recall symptoms fairly accurately [22, 23]. However, recall bias cannot entirely be eliminated. Some may misremember symptom experiences to have occurred within the last 4 weeks due to severity of the symptom. Others who might have experienced mild or transient symptoms or had not found the symptoms to be of any concern may have forgotten about the symptom experience. Therefore, it is possible that individuals with severe GI symptoms and secondary decreased self-rated health/functional capacity might have a higher recall of the symptoms compared to individuals with GI symptoms not affecting their self-rated health/functional capacity.

The symptom-based diagnostic criteria for IBS and FD were based on the Rome III criteria, which have been the accepted gold-standard for diagnosing FGIDs since 2006 [2]. During 2016 Rome IV diagnostic guidelines were published [24] with minor alterations made to the criteria for individual FGIDs. Sood and Ford emphasise in a newly published paper [25], that the most substantial change in the Rome IV is the recognition that a considerable overlap exists between some of the FGIDs and that they should be considered as part of a spectrum, rather than discrete disorders. This is accordance with the findings in the present paper. The alterations should however be kept in mind when comparing future studies.

Another limitation to keep in mind is the fact that we do not have information about the respondents’ comorbidity. Especially among the elderly, it is possible that decreased self-rated health and impaired functional capacity are due to other illnesses and not merely attributable to the GI symptoms. However, the ORs for poor self-rated health and impaired functional capacity increased comparably with the number of symptoms for respondents over and under 50 years of age. Nevertheless, using generic single item questions to measure general self-rated health and functional capacity prevents us from determining the GI symptoms that are the cause of poor self-rated health and impaired functional capacity. In this study, we can merely assess the associations between GI symptoms experience and poor self-rated health and impaired functional capacity.

### Comparison to existing literature

It has previously been demonstrated that GERD, FD and IBS decrease quality of life significantly [4–6]. One of the studies also demonstrated that overlapping symptoms affect bodily pain and general health evaluation [6]. However, that also functional capacity is affected by GI symptoms is new knowledge, and the amplification of the negative effect on both self-rated health and functional capacity in individuals with overlap of symptoms of GERD, FD and IBS gives new insight into the consequences of experiencing multiple GI symptoms.

Associations between functional disorders and anxiety and depression are previously described [26], and an increasing number of studies suggest that persistent and multiple symptoms may lead to severe disability and poor outcome [27]. Patient-reported outcomes (PROs) are increasingly used to improve patient care as a supplement to clinical information [28, 29]. PROs may aid in the early identification of individuals at risk of poor outcome and, with an enhanced focus on the individuals instead of diagnoses, they might prevent harm from unnecessary investigations and treatments inflicted by the healthcare system.

### Implications

Our findings suggest that the experience of multiple GI symptoms is associated with poor self-rated health and impaired functional capacity. Though the symptoms origin from the same organ system and hence might be perceived by the patient as one illness, the negative impact of experiencing symptoms compatible with two or all three symptom complexes, GERD, FD and/or IBS should not be underestimated. As a clinician it is important to consider that symptoms of GERD, FD and/or IBS have a substantial negative impact on the patients’ self-rated health and functional capacity, and furthermore to acknowledge the amplifying effect of overlapping symptoms.

## Conclusion

In conclusion this study demonstrates that individuals experiencing symptoms of either GERD, FD or IBS report significantly low self-rated health as well as impaired functional capacity. This negative effect is amplified in individuals of both gender and all ages experiencing overlapping symptoms of GERD, FD and IBS.

## Additional file

Additional file 1: The Danish Symptom Cohort – a survey about health, symptoms and healthcare-seeking. The questionnaire was not available in hard copy, but for illustrative purposes it has been reproduced in this file. The file contains only items developed for use in this study. The web-based questionnaire contains several leaps based on the answers provided by the respondent (marked with explanatory captions in italic). (DOCX 44 kb)

## Abbreviations

CRS: Danish Civil Registration System
FD: Functional dyspepsia
GERD: Gastroesophageal reflux disease
GI: Gastrointestinal
GP: General Practitioner
IBS: Irritable bowel syndrome
IQR: Interquartile range
OR: Odds ratio
PROs: Patient-reported outcomes
RIIIAQ: ROME III Adult Questionnaire
