# Supplementary material for: Self-rated health and functional capacity in individuals reporting overlapping symptoms of gastroesophageal reflux disease, functional dyspepsia and irritable bowel syndrome - a population based study
Source: BMC Gastroenterol. 2017 May 18;17:65. doi: 10.1186/s12876-017-0622-9 (PMC5437406; doi:10.1186/s12876-017-0622-9)
Supplement: Additional file 1: — The Danish Symptom Cohort – a survey about health, symptoms and healthcare-seeking. The questionnaire was not available in hard copy, but for illustrative purposes it has been reproduced in this file. The file contains only items developed for use in this study. The web-based questionnaire contains several leaps based on the answers provided by the respondent (marked with explanatory captions in italic). (DOCX 44 kb) [file 12876_2017_622_MOESM1_ESM.docx]

*The questionnaire was not available in hard copy, but for illustrative purposes it has been reproduced in this file. The file contains only items developed for use in this study.*

*The web-based questionnaire contains several leaps based on the answers provided by the respondent (marked with explanatory captions in italic).*

| The following questions are about abdominal pain. Furthermore we ask questions regarding various factors that may have impact on abdominal pain and discomfort. |
| --- |

| In **the last 3 months**, how often did you have acid regurgitation or heartburn (a burning epigastric discomfort or burning pain in your chest)? |
| --- |
| - Never - Less than one day a month - One day a month - Two to three days a month - One day a week - More than one day a week - Everyday |

*The next three questions are skipped if the answer is “never” in the above-mentioned questions.*

| When you experience acid regurgitation or heartburn (a burning epigastric discomfort or burning pain in your chest), how severe are your discomforts? |
| --- |
| - Very mild - Mild - Moderately - Severe - Very severe |

| To what extent does your acid regurgitation or heartburn (a burning epigastric discomfort or burning pain in your chest) affect your sleep? |
| --- |
| - My sleep is not affected - My sleep is affected to some extent - My sleep is affected to a great extent |
| To what extent does your acid regurgitation or heartburn (a burning epigastric discomfort or burning pain in your chest) affect your everyday activities? |
| - Not at all - Slightly - Moderately - Quite a bit - Extremely |

| The following questions concern abdominal pain and bowel habits. |
| --- |

*The next questions are related to the Rome 3 criteria for IBS: If the symptoms are experienced less than two to three days a month, the rest of the questions for IBS are skipped*

| In **the last 3 months**, how often did you have discomfort or pain anywhere in your abdomen? | - Never - Less than one day a month - One day a month - Two to three days a month - One day a week - More than one day a week - Every day |
| --- | --- |
| *For women*: Did this discomfort or pain occur only during your menstrual bleeding and not at other times? | - No - Yes - Does not apply because I have had the change in life (menopause) or I am a male |
| Have you had this discomfort or pain **6 months or longer**? | - No - Yes |

| How often did this discomfort or pain get better or stop after you had a bowel movement? | - Never or rarely - Sometimes - Often - Most of the time - Always |
| --- | --- |
| When this discomfort or pain started, did you have more frequent bowel movements? | - Never or rarely - Sometimes - Often - Most of the time - Always |
| When this discomfort or pain started, did you have less frequent bowel movements? | - Never or rarely - Sometimes - Often - Most of the time - Always |
| When this discomfort or pain started, were your stools (bowel movements) looser? | - Never or rarely - Sometimes - Often - Most of the time - Always |
| When this discomfort or pain started, how often did you have harder stools? | - Never or rarely - Sometimes - Often - Most of the time - Always |
| In the **last 3 months**, how often did you have hard or lumpy stools? | - Never or rarely - About 25% of the time - About 50% of the time - About 75% of the time - Always, 100% of the time |
| In the **last 3 months**, how often did you have loose, mushy or watery stools? | - Never or rarely - About 25% of the time - About 50% of the time - About 75% of the time - Always, 100% of the time |

| The following questions concern feeling of fullness after meals and pain or burning sensation in the stomach. |
| --- |

*The next questions are related to the Rome 3 criteria for functional dyspepsia*

| In the **last 3 months**, how often did you feel uncomfortably full after a regular- sized meal? | - Never - Less than one day a month - One day a month - Two to three days a month - One day a week - More than one day a week - Every day |  |
| --- | --- | --- |
| *The next questions only appeared if symptoms are experienced for one day a week or more*  Have you had this uncomfortable fullness after meals **6 months or longer**? | - No - Yes |  |
| In the **last 3 months**, how often were you unable to finish a regular size meal? | - Never - Less than one day a month - One day a month - Two to three days a month - One day a week - More than one day a week - Every day |  |
| Have you had this inability to finish regular size meals **6 months or longer**? | - No - Yes |  |
| In the **last 3 months**, how often did you have pain or burning in the middle of your abdomen, above your belly button but not in your chest? | - Never - Less than one day a month - One day a month - Two to three days a month - One day a week - More than one day a week - Every day |  |
| Have you had this pain or burning **6 months or longer**? | - No - Yes |  |

| We have now finished asking about specific symptoms and discomforts. The next questions are of a general nature and concern your own perception of your health. | |
| --- | --- |
| In general, would you say your health is: |  |
| - Excellent - Very good - Good - Fair - Poor |  |
| Do you feel well enough to do what you feel like doing? |  |
| - Yes, mostly - Yes, sometimes - No, almost never - I don’t know |  |
